# The Good and Bad of β-Catenin in Kidney Development and Renal Dysplasia

**DOI:** 10.3389/fcell.2015.00081

**Published:** 2015-12-22

**Authors:** Felix J. Boivin, Sanjay Sarin, J. Colin Evans, Darren Bridgewater

**Affiliations:** Department of Pathology and Molecular Medicine, McMaster UniversityHamilton, ON, Canada

**Keywords:** kidney development, beta catenin, renal dyspalsia, ureteric epithelium, metanephric mesenchyme, renal stroma

## Abstract

Congenital renal malformations are a major cause of childhood and adult onset chronic kidney disease. Identifying the etiology of these renal defects is often challenging since disruptions in the processes that drive kidney development can result from disruptions in environmental, genetic, or epigenetic cues. β-catenin is an intracellular molecule involved in cell adhesion, cell signaling, and regulation of gene transcription. It plays essential roles in kidney development and in the pathogenesis of renal dysplasia. Here, we review the function of β-catenin during kidney development and in the genesis of renal dysplasia.

## Introduction

Renal dysplasia is a developmental disorder of the kidney that affects 1 in 250 live births (N'Guessen et al., 1984; Pohl et al., 2002). This disorder can lead to childhood renal failure, adult onset chronic renal insufficiency, and hypertension. Currently there are no cures for this disease and treatment is limited to managing the symptoms. This is due, in part, to a lack of understanding of the mechanisms of the pathogenesis of renal dysplasia. During normal kidney development β-catenin is expressed in the ureteric epithelium, metanephric mesenchyme, and renal stroma. Within these cell populations β-catenin modulates genetic programs that are essential for the control of branching morphogenesis and nephrogenesis. In human dysplastic kidneys, β-catenin is overexpressed in the ureteric epithelium, metanephric mesenchyme, and renal stroma suggesting a pathogenic role in renal dysplasia (Hu et al., 2003; Sarin et al., 2014). By manipulating the levels of β-catenin in animal models, studies have provided significant insight into the normal and pathogenic roles for β-catenin in kidney formation and the genesis of renal dysplasia. Here we review the normal developmental functions of β-catenin in the different cell lineages of the kidney and the contribution of β-catenin to renal dysplasia.

## Anatomy of mammalian kidney development

**Figure 1:**
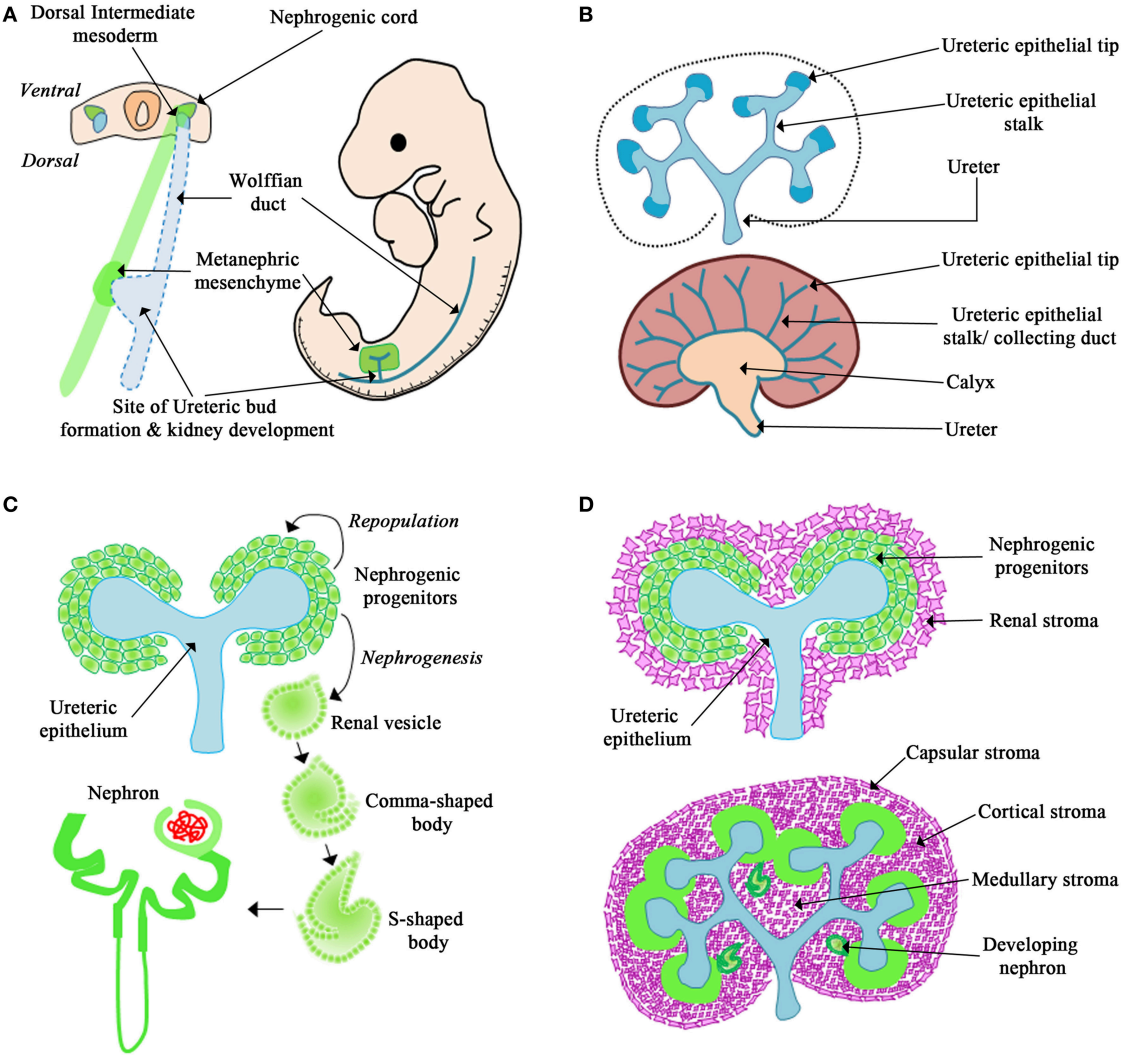
**Overview of kidney development. (A)** The Wolffian duct originates from the dorsal intermediate mesoderm. The metanephric mesenchyme originates from the nephrogenic cord. The Wolffian duct forms an outgrowth called the ureteric bud that migrates into the surrounding mesenchyme and undergoes the first dichotomous branching morphogenesis event. **(B)** The ureteric epithelium is composed of the ureteric tip and stalk region. In response to signals from the mesenchyme, the tips of the ureteric epithelium undergo continued branching morphogenesis and the stalks will differentiate to form the collecting duct system, renal calyx, and ureter. **(C)** In response to signals from the ureteric epithelium the mesenchyme forms a cap around the ureteric tip. This cap mesenchyme will differentiate into nephrons or repopulate the nephrogenic progenitors. **(D)** The renal stroma surrounds the nephrogenic progenitors and will differentiate to form the capsular, cortical, and medullary stroma.

Metanephric kidney development is dependent upon the interactions of three main cell types: the ureteric epithelium, the metanephric mesenchyme, and the renal stroma. The formation of the mammalian kidney begins at embryonic day (E) 9.5 in the mouse when epithelial cells emerge from the intermediate mesoderm (termed the Wolffian duct) and migrate caudally toward the urogenital sinus (Figure 1A). At E10.5 in the mouse and 5 weeks gestation in humans an outgrowth of the caudal portion of the Wolffian duct, termed the ureteric bud, migrates into the adjacent metanephric mesenchyme (Figure 1A). In response to signals from the metanephric mesenchyme, the ureteric epithelium undergoes the first branching event to form a T-shaped branch at E11.5 in the mouse (Figure 1A; Saxén and Sariola, 1987). Each ureteric bud tip subsequently undergoes reiterative cycles of elongation, bifurcation, and differentiation to give rise to the metanephric collecting duct system, renal calyces, and ureter (Figure 1B). This process is known as branching morphogenesis and continues for 10 cycles to form ~1500 collecting ducts in mice (Cebrián et al., 2004) and 15 cycles to form 60,000 collecting ducts in humans (Saxén and Sariola, 1987).

Simultaneously, the metanephric mesenchyme receives signals from the ureteric epithelium, and responds by tightly clustering around the ureteric bud tips. These clusters of condensed mesenchyme form the nephrogenic progenitors, which are populations of cells destined to form the nephron (Figure 1C). Depending on the molecular cues from the ureteric epithelium, the nephrogenic progenitors either undergo self-renewal to repopulate the condensed mesenchyme or differentiate into epithelial cells (Little and McMahon, 2012). The newly formed nephrogenic epithelial cells will then undergo nephrogenesis, the process to form the nephron via a series of distinct morphological changes (i.e., renal vesicle, comma-shaped, and s-shaped bodies) to form the nephron in a process termed nephrogenesis. This process will give rise to 10,000 nephrons (Cebrián et al., 2004) in the mouse and 200,000–1.8 million nephrons in humans (Saxén and Sariola, 1987; Hughson et al., 2003; Cebrián et al., 2004; Cain et al., 2010).

Shortly after the invasion of the ureteric bud into the metanephric mesenchyme a third cell population, termed the renal stroma, is observed surrounding the condensed mesenchyme (Figure 1D; Hatini et al., 1996; Cullen-McEwen et al., 2005; Li et al., 2014). The renal stroma is a population of matrix-producing fibroblast cells that surround adjacent nephrogenic structures and collecting ducts (Li et al., 2014). Studies have demonstrated that the renal stroma cell population is required for kidney development by modulating branching morphogenesis and nephrogenesis (Das et al., 2013; Hum et al., 2014; Li et al., 2014; Boivin et al., 2015). However, the mechanism of how the renal stroma regulates kidney development is only beginning to be understood.

The impairment of the processes that guide branching morphogenesis and nephrogenesis during kidney development can result in reduced nephron number and abnormal collecting duct formation, which can increase the risk of developing childhood renal failure or adult onset chronic renal insufficiency.

## The traditional and non-traditional roles of β-catenin

**Figure 2:**
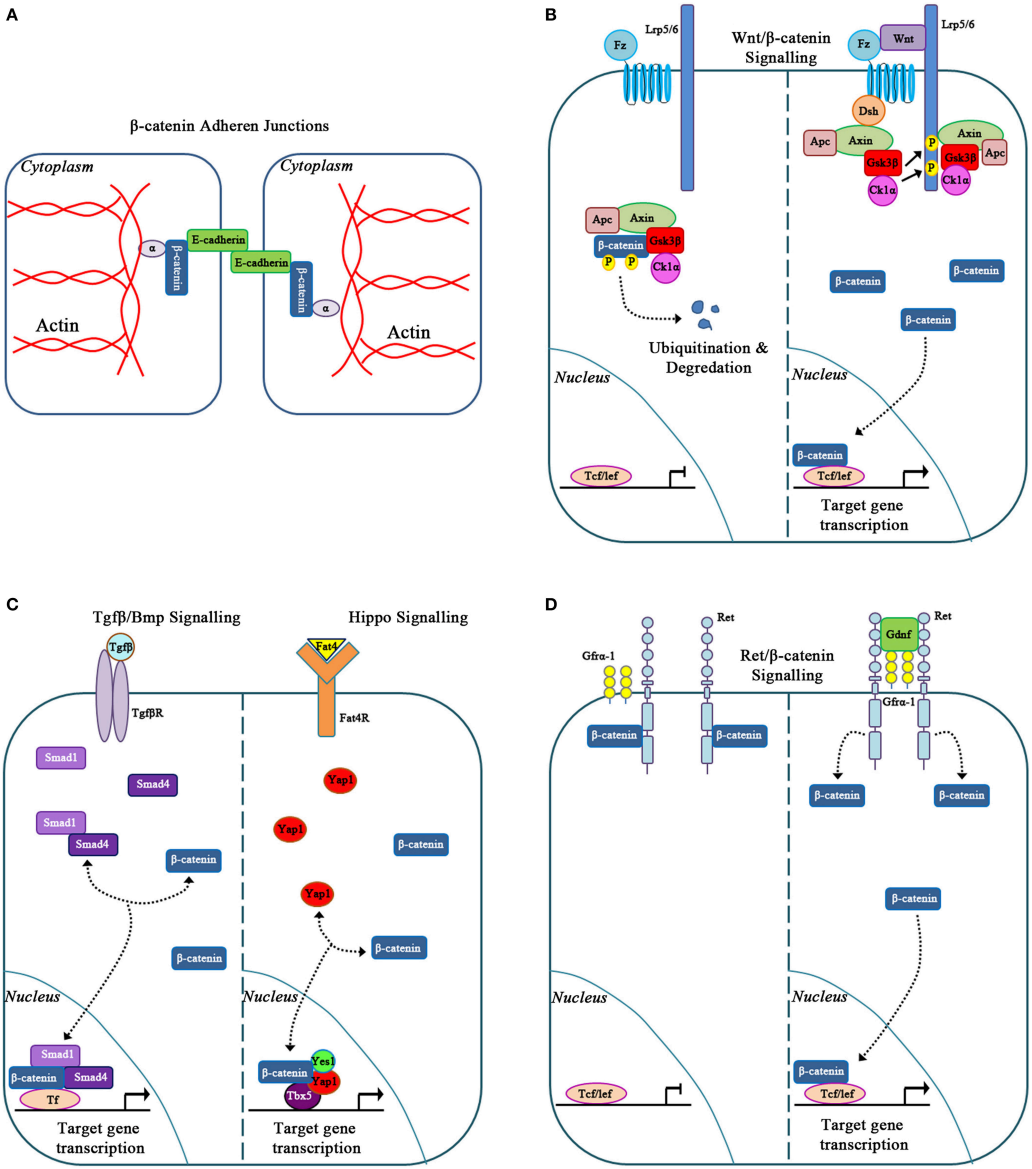
**Classic and novel functions for β-catenin. (A)** β-catenin in cell to cell adhesion via adherens junctions. **(B)** β-catenin is a signaling molecule involved in Canonical Wnt signaling. **(C)** β-catenin forms novel molecular transcriptional complexes with Tgfβ/Bmp and Hippo signaling effectors. **(D)** β-catenin signals in response to Gdnf/Ret signaling.

β-catenin is an evolutionarily conserved multi-functional protein involved in various cellular activities depending on its intracellular localization (Heuberger and Birchmeier, 2010). At the cell membrane, β-catenin binds to the intracellular domain of E-cadherin to form adherens junctions between adjacent epithelial cells. Within these junctions β-catenin binds to α-catenin to bridge E-cadherin to the actin cytoskeleton, a process that is required for cell movements during morphogenesis (Figure 2A; Gumbiner, 2000, 2005). β-catenin is also involved in canonical Wnt signaling (Clevers and Nusse, 2012; Figure 2B). In this pathway, the absence of a Wnt signal results in the recruitment of cytoplasmic β-catenin by a destruction complex that is composed of axin, adenomatosis polyposis coli (APC), glycogen synthase kinase 3 (Gsk3β) and casein kinase 1α (Ck1α; Logan and Nusse, 2004). This destruction complex binds and phosphorylates β-catenin at serine/threonine residues leading to ubiquitination and proteasomal degradation of β-catenin (Figure 2B). In the presence of a Wnt signal, Wnt ligands bind to the Frizzled and LRP5/6 co-receptor complex, which results in the phosphorylation of disheveled (Dsh). Phosphorylated Dsh sequesters the destruction complex to the cell membrane and away from β-catenin. As a result, β-catenin is not phosphorylated by the destruction complex and accumulates in the cytoplasm. β-catenin can then translocate to the nucleus where it binds to the TCF/LEF family of DNA-bound co-transcriptional activators to regulate gene transcription (Figure 2B; Zeng et al., 2008; MacDonald et al., 2009). Activation of this canonical Wnt/β-catenin mediated signaling pathway regulates several genes involved in cell proliferation, cell fate specification, and differentiation (Logan and Nusse, 2004).

While traditionally involved in canonical Wnt signaling, β-catenin is also involved within other signaling pathways. In the study of Bmp signaling during kidney development and disease, β-catenin interacted with Smad1 and Smad4. Together these proteins form a transcriptional complex (Hu et al., 2003) that controls c-myc, which then regulates kidney epithelial tubule proliferation and differentiation (Hu and Rosenblum, 2005). The molecular interactions between β-catenin and Smad proteins downstream of the Bmp and Tgf-β signaling pathways also control the expression of genes, such as Xtwn and Msx2, which are involved in cell fate determination in other organ systems and may also be important in the kidney (Labbé et al., 2000; Hussein et al., 2003).

More recent studies have demonstrated that β-catenin can interact with members of the Hippo signaling pathway to control organ size (Figure 2C). In the developing heart, nuclear β-catenin forms a transcriptional complex with the Hippo signaling effector YAP to regulate cardiomyocyte proliferation (Heallen et al., 2011). Similarly, β-catenin also forms a molecular complex with YAP, YES1, and TBX5 to regulate proliferation in various β-catenin-driven human cancer cell lines (Rosenbluh et al., 2012). Specifically in the condensed mesenchyme of the kidney, deletion of the Hippo signaling effectors Taz and Yap results in reduced expression of β-catenin target genes, *Amph, Tafa5, Pla2g7*, and *Cited1*, thus decreasing the nephrogenic progenitor cell population (Das et al., 2013). This suggests that β-catenin and the Hippo signaling effectors interact to modulate the nephrogenic progenitor cell population. Furthermore, β-catenin forms a transcriptional complex with Yap, Yes1, and Tbx5 and binds to the promoter region of anti-apoptotic gene *bcl2l1* in the developing kidney (Boivin and Bridgewater, 2014). However, this novel transcriptional complex's biological outcomes in the specific cells of the developing kidney remain to be explored.

β-catenin is also involved in the Gdnf/Ret signaling pathway. Gdnf is expressed by the condensing mesenchyme cells, and its receptor, Ret, localizes to the ureteric epithelium. Activation of the Ret receptor initiates ureteric bud outgrowth from the nephric duct and is essential for continued branching morphogenesis. Recent studies demonstrate that β-catenin interacts with the Ret receptor during kidney development. Upon stimulation of the Ret receptor by Gdnf, β-catenin is released from the cytoplasmic tail of Ret and migrates to the nucleus of ureteric epithelial cells (Sarin et al., 2014; Figure 2D). It is likely that this activation of β-catenin via Gdnf/Ret signaling regulates unique genetic targets to control specific biological processes during normal kidney development and kidney disease. This concept is supported by studies in thyroid carcinomas in which Ret/β-catenin signaling regulates *cyclinD1, Egr1*, and *JunB* to promote cell proliferation (Gujral et al., 2008). Altogether, these studies demonstrate that β-catenin is a promiscuous molecule, interacting and communicating within numerous signaling pathways and transcription factors to modulate specific biological processes.

## β-catenin in normal kidney development

During kidney development, β-catenin demonstrates a specific spatial and temporal expression pattern. In the embryonic mouse and human kidney, β-catenin is expressed in the ureteric epithelium, in all stages of the developing nephron, and in the renal stroma (Bridgewater et al., 2008; Sarin et al., 2014; Boivin et al., 2015). The cytoplasmic and nuclear expression patterns within these cells suggest functional roles in cell adhesion, cell signaling, and regulation of gene transcription. The analysis of transgenic mice containing *Tcf/Lef* binding sites upstream of a β-galactosidase reporter transgene confirmed β-catenin-mediated transcriptional activity in the Wolffian duct, ureteric epithelium, developing nephrons, and renal stroma (Iglesias et al., 2007; Bridgewater et al., 2008; Yu et al., 2009). Conditional mouse models have been used to greatly advance our understanding of the specific functional roles of β-catenin in the different cell lineages that are necessary for kidney development. These mouse models will be reviewed below.

### β-catenin in the Wolffian duct and ureteric epithelium

One of the earliest known roles for β-catenin in kidney development is in the maintenance of *Gata3* expression within the Wolffian duct (Grote et al., 2008). *Gata3* controls proliferation and caudal extension of the Wolffian duct epithelium toward the urogenital sinus (Grote et al., 2006). *In silico* analysis of the Gata3 promoter region and β-galactosidase reporter assays demonstrated that Gata3 is a direct downstream transcriptional target of β-catenin (Grote et al., 2008). The targeted deletion of β-catenin from the Wolffian duct resulted in a loss of *Gata3* expression and failure to initiate normal ureteric budding, which ultimately leads to renal agenesis (Grote et al., 2008). In addition to β-catenin's effect on Wolffian duct elongation, β-catenin also plays essential roles in ureteric epithelial branching morphogenesis and differentiation (Bridgewater et al., 2008; Marose et al., 2008). Targeted inactivation of β-catenin in the ureteric lineage resulted in decreased expression of Emx2, a transcription factor essential in the maintenance of ureteric bud tip cells and branching morphogenesis (Miyamoto et al., 1997). This loss of Emx2 expression leads to reductions in its downstream targets *Lim1, c-Ret, Pax2*, and *Wnt11*, resulting in reduced branching morphogenesis and renal hypoplasia (Miyamoto et al., 1997; Bridgewater et al., 2008). The transcriptional control was likely direct in nature since the *Emx2* promoter contains numerous *Tcf/Lef* consensus binding sequences (Theil et al., 2002). Additionally, *Emx2* deficient mice exhibit branching defects and hypoplastic kidneys similar to the phenotype observed in mice deficient for β-catenin in ureteric epithelial cells (Miyamoto et al., 1997). Together, these studies support a role for β-catenin in regulating branching morphogenesis via the control of *Emx2* expression.

β-catenin is also required for proper ureteric epithelium differentiation. During the reiterative cycles of branching morphogenesis, the ureteric epithelium is spatially organized into the ureteric tip and stalk regions (Figure 1B). Each region displays distinct gene expression patterns (Bridgewater and Rosenblum, 2009) that are necessary for the development of the collecting duct system (Costantini, 2012). Marose et al. demonstrated that a loss of β-catenin from the ureteric epithelium leads to the activation of aquaporin-3 and ZO-1α+ in the ureteric bud tips. These genes are normally associated with the fully differentiated collecting duct system (Marose et al., 2008). Similarly, Bridgewater et al. demonstrated mice with a β-catenin deficiency in the ureteric epithelium exhibited a loss of ureteric bud tip associated genes. This led to a loss of tip cell identity and resulted in reduced branching morphogenesis (Bridgewater et al., 2008). Taken together, these studies demonstrate that β-catenin regulates a hierarchy of gene expression in the ureteric epithelium and maintains the ureteric bud tip identity, thus making the ureteric epithelium permissive for branching morphogenesis.

Considering the importance of β-catenin in cell adhesion and epithelial morphogenesis (Baum and Georgiou, 2011), two independent studies (Bridgewater et al., 2008; Marose et al., 2008) analyzed the effects of the loss of β-catenin in the ureteric epithelium on adherens junctions. These studies demonstrated that the formation and maintenance of adherens junctions were unaffected by the loss of β-catenin, likely due to the compensatory function of γ-catenin (Zhurinsky et al., 2000). These studies support the hypothesis that defects in branching morphogenesis result primarily from β-catenin mediated transcription (Bridgewater et al., 2008; Marose et al., 2008). However, the assembly and disassembly of adherens junctions is required for the complex movements of epithelial tubes during morphogenesis (Baum and Georgiou, 2011) and it would be interesting to determine if the dynamic nature of adherens junctions is maintained in the absence of β-catenin.

### β-catenin in the mesenchyme

β-catenin plays essential roles in the induction of the condensed mesenchyme. This role was first demonstrated in *Wnt9b* deficient mice. During normal kidney development, Wnt9b, which is secreted from the ureteric epithelium, activates the canonical Wnt/β-catenin signaling pathway in the neighboring condensed mesenchyme cells. This inductive β-catenin mediated signal then up-regulates Wnt4 gene expression (Carroll et al., 2005; Park et al., 2007). Wnt4 activates downstream genetic targets *Fgf8, Pax8*, and *Lhx1* in a β-catenin dependent manner, which is essential for mesenchymal-to-epithelial transition (MET) and the formation of pre-tubular aggregates (Stark et al., 1994; Kispert et al., 1998; Park et al., 2007). The importance of β-catenin is also highlighted by the genetic deletion of β-catenin specifically in the nephrogenic progenitors. This resulted in reduced expression of early developing nephron markers *Fgf8, Pax8, Wnt4*, and *Lhx1* leading to stalled nephrogenic structures and markedly reduced nephrogenesis (Park et al., 2007). Furthermore, the sustained overexpression of β-catenin in the nephrogenic progenitors in *Wnt9b* deficient mice, which exhibit an absence of *Fgf8, Wnt4*, and *Lhx1*, resulted in the rescue of the expression of these nephrogenic genes (Park et al., 2007). This demonstrates β-catenin in the nephrogenic progenitors acts downstream of both Wnt9b and Wnt4 to initiate the nephrogenic program. While β-catenin signaling is sufficient to initiate nephrogenesis, it must be down-regulated for nephrogenesis to proceed to the renal vesicle stage (Park et al., 2007).

The deletion of β-catenin in the condensed mesenchyme results in the depletion of the nephrogenic progenitors thus supporting a role for β-catenin in the maintenance or self-renewal of this cell population (Karner et al., 2011; Sarin et al., 2014). In the search for β-catenin targets involved in the maintenance of nephrogenic progenitors, Karner and colleagues demonstrated that β-catenin and Six2, a transcription factor essential for self-renewal of the nephrogenic progenitors (Kobayashi et al., 2008), work together to regulate the expression of genes in the nephrogenic progenitors (Karner et al., 2011). These candidate β-catenin target genes, among others, include *Cited1, Tafa5, Pla2g7*, and *Gdnf* (Karner et al., 2011). Their direct regulation by β-catenin is supported by co-immunoprecipitation and chromatin immunoprecipitation studies that demonstrate β-catenin forms a molecular complex with Six2 to bind to the promoter regions of nephrogenic progenitor genes, such as *Gdnf* (Park et al., 2012; Sarin et al., 2014). However, functional studies to determine if β-catenin and Six2 regulate the expression of all these genes have yet to be performed.

While investigating the cooperative role between Six2 and β-catenin, distinct gene expression domains within the condensed mesenchyme were identified. Park and colleagues demonstrated that Six2 and the Wnt9b/β-catenin signaling form gradients in the condensed mesenchyme to balance the proliferation and induction of the nephrogenic progenitors (Park et al., 2012). The condensed mesenchyme cells farthest from the ureteric epithelium receive a low Wnt9b signal, which results in low levels of β-catenin activity. Six2 is then able to repress β-catenin's transcriptional activity leading to self-renewal of the nephrogenic cell population. Conversely, the cells closest to the ureteric epithelium receive a high Wnt9b signal resulting in increased β-catenin activity. This leads to the initiation of nephrogenesis, activation of Wnt4, and MET (Park et al., 2012). This analysis demonstrates that the tight control of β-catenin activity is essential for balancing self-renewal of the nephrogenic progenitors and induction of nephrogenesis. It also suggests distinct zones within the nephrogenic progenitors that exhibit varying levels of β-catenin activity. Future studies are required to elucidate the specific β-catenin genetic targets and their functions within these distinct domains.

### β-catenin in the renal stroma

As kidney development progresses, the stromal population divides into three distinct populations: capsular, cortical, and medullary stroma. Within these three stromal populations the intracellular localization of β-catenin is variable, likely due to unique functional roles within each stromal cell sub-population (Boivin et al., 2015). In the renal capsule, β-catenin localizes primarily to the cell membrane. Since the integrity of the renal capsule is essential to maintain the high pressures within the kidney parenchyma, its primary role is likely involved in forming cell-cell junctions (Garcia-Estañ and Roman, 1989). Studies have also demonstrated that capsular stromal cells communicate with underlying cell populations, such as the cortical stroma and condensed mesenchyme (Levinson et al., 2005; Yallowitz et al., 2011). Mutant mice that lack β-catenin in stromal cells demonstrate a paucity of renal capsule with loosely packed capsular cells that are non-adherent to the underlying parenchyma (Boivin et al., 2015). These studies support a role for β-catenin in capsular cell-cell adhesion and capsular adhesion to the underlying cells. Further, these capsular defects likely contribute to disrupted communication with the cortical stroma and condensed mesenchyme. Thus, further investigation of β-catenin's role with respect to capsular stromal cell integrity is required to determine how it modulates cellular functions in the underlying cells.

The nuclear and cytoplasmic localization of β-catenin in the cortical stroma, which is directly adjacent to the condensed mesenchyme, suggests β-catenin may modulate cell signaling and gene expression to regulate nephrogenesis. In support of this theory, mutant mice with stromal β-catenin deficiency demonstrate a single layer of loosely packed condensed mesenchyme compared to *WT* kidneys, which exhibit 3–4 cell layers (Boivin et al., 2015). These alterations were caused by reduced levels of *Wnt9b* in the ureteric epithelium resulting in reduced expression of Wnt9b/β-catenin dependent genes (*Tafa5, Cited1*, and *Amph*) in the nephrogenic progenitor cells. These data suggest that β-catenin in the cortical stroma controls gene expression in neighboring cell populations possibly through direct cell-cell interactions or by β-catenin regulating secreted factors from the renal stroma. These possibilities are supported by studies that demonstrated renal stromal cells intermingle with the condensed mesenchymal cells and make direct contacts with the ureteric epithelium (Schnabel et al., 2003; Boivin et al., 2015). There is also evidence supporting stromal secreted factors signaling to the ureteric epithelium or mesenchyme to modulate gene transcription (Li et al., 2014). Future studies will be required to determine the identity of the factors controlled by β-catenin, the role of stromal cells interacting with ureteric epithelium, and the specific mechanisms of the stromal cells to modulate gene expression in the neighboring cells.

In medullary stromal cells, β-catenin primarily localizes to the nucleus (Boivin et al., 2015) and seems to play a prominent role in the regulation of gene transcription (Yu et al., 2009). The specific deletion of β-catenin in all stromal cells results in defects in cortico-medullary axis formation and a loss of medullary stroma (Yu et al., 2009). Yu et al propose that Wnt7b, secreted from the ureteric epithelium, signals to the medullary stroma and activates a β-catenin mediated signaling pathway that controls proper cortico-medullary patterning and contributes to epithelial tubule elongation (Yu et al., 2009; Maezawa et al., 2012; Boivin et al., 2015). While the specific genetic targets activated by Wnt7b/β-catenin signaling in the medullary stroma are not well defined, one possible target is the cyclin-dependent kinase inhibitor p57Kip2, which is markedly reduced in both *Wnt7b* and β-catenin stromal cell mutants (Yu et al., 2009). This suggests that β-catenin in the medullary stroma regulates genetic programs that control medullary stromal cell development and/or maintenance. Taken together, these studies highlight essential roles for β-catenin in the renal stroma.

## β-catenin in renal dysplasia

Renal dysplasia is a congenital kidney malformation that affects up to 0.1% of live births and has an even higher prevalence of 4% in fetus and infants at autopsy (Chen and Chang, 2015). The label “dysplasia” is given to kidneys with structural abnormalities that include, at the gross level, renal agenesis, hypoplasia, hypodysplasia, multiplex kidneys with duplicate ureters, and multicystic dysplastic kidneys (Woolf et al., 2004; Winyard and Chitty, 2008; Goodyer, 2009). At the histopathological level, dysplastic kidneys exhibit defects in cortical and medullary patterning (Piscione and Rosenblum, 1999), disorganization of the collecting system and nephron elements (Woolf et al., 2004), dilated/cystic epithelial tubules (Hu et al., 2003; Katabathina et al., 2010; Trnka et al., 2010), undifferentiated tubules and mesenchyme (Winyard and Chitty, 2008), cystic glomeruli (Sanna-Cherchi et al., 2007), and expanded loosely arranged stroma (Woolf et al., 2004; Winyard and Chitty, 2008). These abnormalities can be diffuse (involving the entire kidney), segmental (involving segments of the kidney) or focal (affected regions intermingled with normal tissue; Winyard and Chitty, 2008). Defects in the processes that guide kidney development are the major contributing factors leading to the gross and histopathological changes observed in renal dysplasia.

Despite our growing understanding of renal dysplasia, the precise causes and mechanisms contributing to the pathogenesis remain poorly understood. The abnormal expression of numerous growth factors, signaling pathways, and transcription factors has been observed in humans and mouse models of renal dysplasia (Jain et al., 2007; Bridgewater et al., 2011; Thomas et al., 2011). Furthermore, studies have linked specific mutations in genes such as Sal-like 1 (*Sall1*), Paired Box gene 2 (*Pax2*), and Transcription factor 2 (*TCF2*) to the genesis of renal dysplasia (Weber et al., 2006). The overexpression of β-catenin in both human renal dysplastic tissue with different underlying etiologies (Hu et al., 2003; Sarin et al., 2014) and in numerous mouse models of renal dysplasia (Bridgewater et al., 2011; Sarin et al., 2014), strongly suggest that overexpression of β-catenin is a contributing factor to the pathogenesis of renal dysplasia. The use of conditional mouse models and human dysplastic kidney tissue has greatly advanced our understanding of the pathogenic role of β-catenin overexpression (Hu et al., 2003; Sarin et al., 2014) and will be reviewed below.

### β-catenin overexpression in the ureteric epithelium

Hu et al. demonstrated that β-catenin is overexpressed in ureteric epithelial derived structures in dysplastic human kidney tissue, and this was the first finding to highlight dysregulated β-catenin expression in human renal dysplasia (Hu et al., 2003). The functional role of β-catenin overexpression in the ureteric epithelium was initially investigated by creating a mouse model with targeted overexpression of β-catenin specifically in the ureteric epithelium (Bridgewater et al., 2011). These mutant mice exhibited severe renal hypodysplasia characterized by marked defects in branching morphogenesis and nephrogenesis (Bridgewater et al., 2011). In this transgenic model, a mechanism was defined in which β-catenin overexpression led to the upregulation of Tgfβ2 and the Wnt inhibitor *Dkk1* specifically in the ureteric epithelium. The overexpression of Tgfβ2 in epithelial cells inhibited ureteric branching and caused ectopic and premature differentiation of nephrogenic progenitors, while *Dkk1* inhibited Wnt4 activity (Bridgewater et al., 2011). In a similar study, Marose et al. demonstrated that ureteric-specific β-catenin overexpression in mice prevented ureteric epithelium differentiation to form collecting ducts resulting in hypodysplastic cystic kidneys (Marose et al., 2008). Together, these studies established that β-catenin overexpression results in marked alterations in branching morphogenesis and nephrogenesis due to disrupted gene expression in the ureteric epithelium.

### β-catenin overexpression in the metanephric mesenchyme

**Figure 3:**
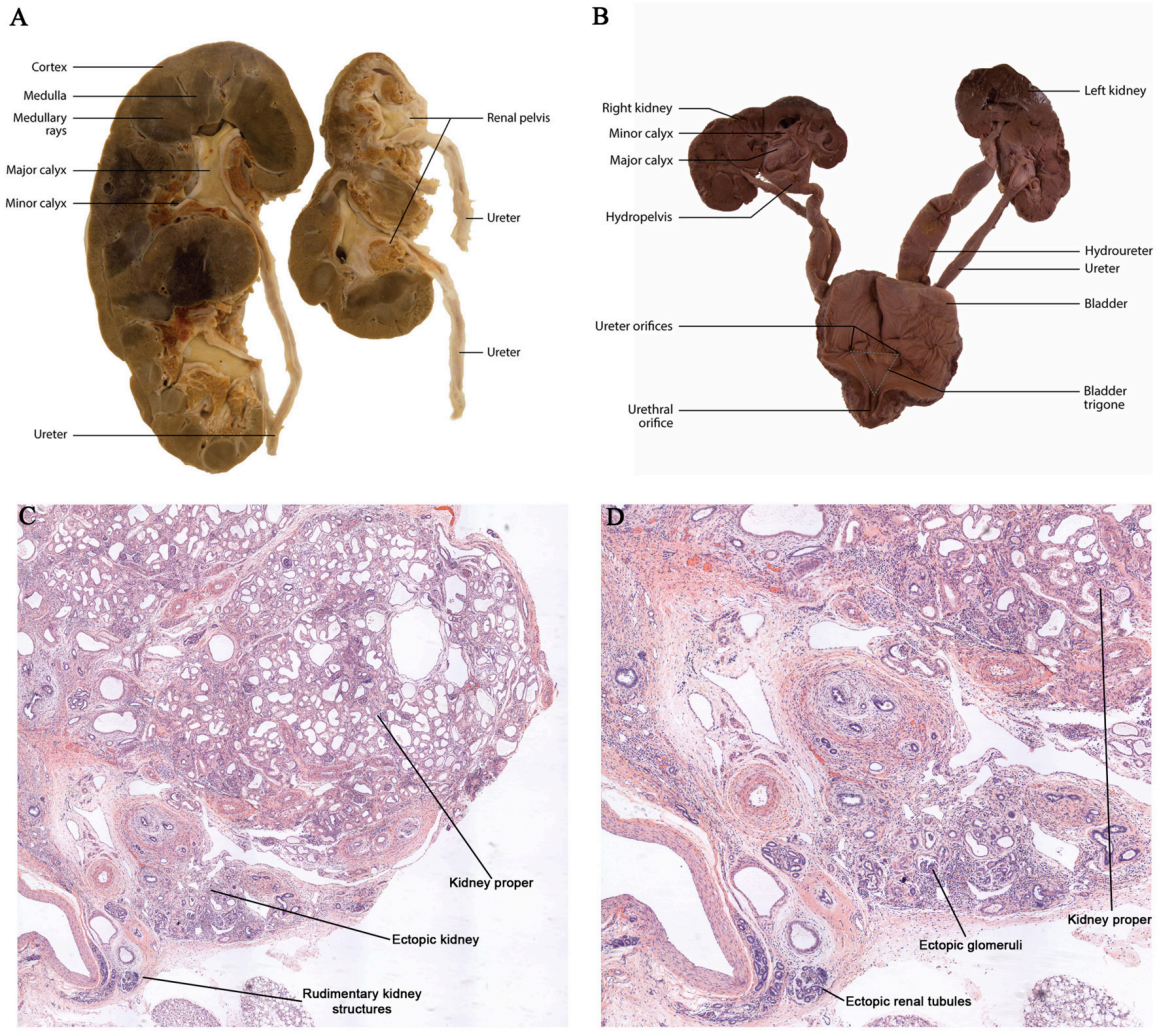
**Dysplastic multiplex kidneys. (A,B)** Gross anatomy of post-natal and embryonic multiplex kidneys with bilateral bifid ureters. **(C,D)** Histological analysis of dysplastic kidneys demonstrating multiplex kidneys.

In addition to β-catenin overexpression in ureteric epithelial cells, human dysplastic renal tissue also demonstrates β-catenin overexpression in the mesenchymal and stromal cells (Sarin et al., 2014). A close examination of the expression pattern showed that β-catenin was primarily increased in the mesenchymal and stromal cell nuclei suggesting a role in the regulation of gene transcription (Sarin et al., 2014). Park et al. demonstrated, using the *Six2-Cre* mice to overexpress β-catenin in the nephrogenic progenitors, an inability to form renal vesicles resulting in severely hypodysplastic kidneys (Park et al., 2007). Analysis of a mouse model using the *RAR*β*2Cre* (Kobayashi et al., 2005) to overexpress β-catenin in mesenchyme demonstrated histopathological characteristics consistent with human renal dysplasia, including the presence of large cortical and medullary cysts, undifferentiated mesenchyme, cystic glomeruli, expanded stromal cells surrounding dilated tubules, and multiplex kidneys containing numerous rudimentary kidney structures (sometimes up to eight per animal; Park et al., 2007; Sarin et al., 2014; Figures 3C,D). The multiplex kidneys observed in the mouse model resulted from β-catenin in the mesenchyme upregulating *Gdnf* expression and consequently the ectopic ureteric budding off the Wolffian duct (Sarin et al., 2014). This *Gdnf* overexpression was also observed in human fetal and postnatal dysplastic kidney tissue, specifically in the mesenchyme and on the surface of the ureteric epithelium. Interestingly, the analysis of human dysplastic tissue from the McMaster University Anatomy Program's pathology library revealed several cases of human renal dysplasia with multiplex kidneys, supporting the accuracy of the mouse model to the human condition (Sarin et al., 2014; Figures 3A,B). These studies demonstrate that mesenchymal overexpression of β-catenin disrupts branching morphogenesis and nephrogenesis by misregulating important kidney development genes in human and mouse models of renal dysplasia.

### β-catenin overexpression in the kidney stroma

A notable histopathological feature of renal dysplasia is an expansion of the stroma cell population (Woolf et al., 2004; Winyard and Chitty, 2008). The analysis of β-catenin expression in human dysplastic kidneys revealed β-catenin is markedly increased, primarily in the nucleus of the expanded stromal population (Sarin et al., 2014). While the molecular mechanisms for an overexpression of stromally expressed β-catenin in the pathogenesis of renal dysplasia is not yet known, studies have provided some evidence to suggest a potential role (Maezawa et al., 2012; Sarin et al., 2014). In the embryonic mouse kidney the overexpression of β-catenin exclusively in stromal progenitors is sufficient to cause severe kidney developmental abnormalities. These include several characteristics consistent with renal dysplasia such as disrupted branching morphogenesis, reduced nephrogenesis, and an expansion of the stromal population (Boivin et al., 2015). Further evidence is provided in a mouse model that overexpresses β-catenin in both the condensed mesenchyme and stroma (Maezawa et al., 2012; Sarin et al., 2014). The kidney phenotype in these two models exhibited large, misshapen lobular dysplastic kidneys at birth (Maezawa et al., 2012; Sarin et al., 2014). Conversely, mice with β-catenin overexpressed exclusively in the nephrogenic progenitors result in renal agenesis at birth (Park et al., 2007). Therefore, the marked differences in the kidney phenotypes are likely due to the functional contributions of β-catenin in the renal stroma.

Congenital obstructive nephropathy includes myofibroblast transformation and interstitial fibrosis culminating in renal dysplasia (Nagata et al., 2002). The overexpression of β-catenin in postnatal medullary stroma was sufficient to promote interstitial fibrosis through an upregulation of α-smooth muscle actin and myofibroblast transformation (DiRocco et al., 2013). Considering β-catenin's role in postnatal fibrosis, it is likely that stromal β-catenin is also a central player in interstitial fibrosis observed in renal dysplasia caused by congenital obstructive nephropathy. Taken together, these studies highlight potential roles for β-catenin in the renal stroma in the pathogenesis of renal dysplasia.

## Conclusion

Currently there are no cures for renal dysplasia and treatment is limited to managing the symptoms. The literature reveals β-catenin is a central player in kidney development and the pathogenesis of renal dysplasia. β-catenin is involved in regulating novel signaling pathways, transcriptional complexes, and genes that contribute to renal dysplasia. The continued investigation of β-catenin in kidney development and disease will undoubtedly identify novel signaling pathways and transcriptional targets for preventative and therapeutic treatments.

## Funding

This work was funded by NSERC (405644), Kidney Foundation of Canada, and CIHR (142264).

### Conflict of interest statement

The authors declare that the research was conducted in the absence of any commercial or financial relationships that could be construed as a potential conflict of interest.
